# A Novel Method to Increase LinLog CMOS Sensors’ Performance in High Dynamic Range Scenarios

**DOI:** 10.3390/s110908412

**Published:** 2011-08-29

**Authors:** Antonio Martínez-Sánchez, Carlos Fernández, Pedro J. Navarro, Andrés Iborra

**Affiliations:** 1 Supercomputing and Algorithms Group, CSIC-UAL Associated Unit, University of Almeria, 04120 Almeria, Spain; E-Mail: anmartinezs@ual.es; 2 DSIE, Universidad Politécnica de Cartagena, Campus Muralla del Mar, s/n. E-30202 Cartagena, Spain; E-Mails: pedroj.navarro@upct.es (P.J.N.); andres.iborra@upct.es (A.I.)

**Keywords:** adaptive control, HDR imaging, image sensors, LinLog CMOS sensor, outdoor vision

## Abstract

Images from high dynamic range (HDR) scenes must be obtained with minimum loss of information. For this purpose it is necessary to take full advantage of the quantification levels provided by the CCD/CMOS image sensor. LinLog CMOS sensors satisfy the above demand by offering an adjustable response curve that combines linear and logarithmic responses. This paper presents a novel method to quickly adjust the parameters that control the response curve of a LinLog CMOS image sensor. We propose to use an Adaptive Proportional-Integral-Derivative controller to adjust the exposure time of the sensor, together with control algorithms based on the saturation level and the entropy of the images. With this method the sensor’s maximum dynamic range (120 dB) can be used to acquire good quality images from HDR scenes with fast, automatic adaptation to scene conditions. Adaptation to a new scene is rapid, with a sensor response adjustment of less than eight frames when working in real time video mode. At least 67% of the scene entropy can be retained with this method.

## 1. Introduction

**Figure 1.**
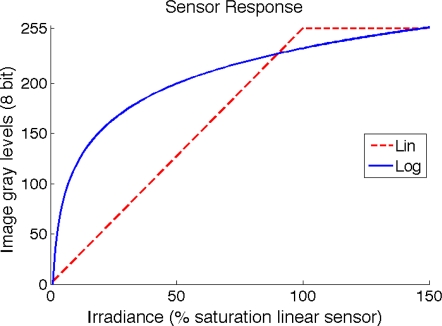
Two typical sensor responses: linear response (red) and logarithmic response (blue). Adjusting the sensor response to a logarithmic curve is a good strategy for increasing the dynamic range.

**Figure 2.**
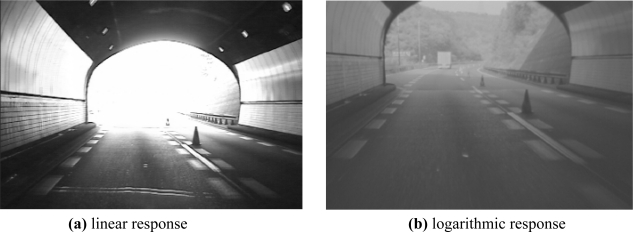
A logarithmic response improves the brighter areas of a scene, but reduces the contrast. Source: OMROM.

The dynamic range (DR) of an image sensor defines the relation between the minimum and maximum light that it can detect [1]. Broadly speaking, the dynamic range for a CCD/CMOS sensor represents its capacity to retain scene information from both highly lighted and shaded scenes. Common CCD/CMOS sensors present a linear response to scene irradiance. More advanced sensors try to increase the dynamic range by converting the linear response to a logarithmic-like response, as shown in Figure 1, providing image enhancement [2] as shown in Figure 2.

A high dynamic range is of major importance for computer vision systems that work with images taken from outdoor scenes—traffic control [3], security surveillance systems [4], outdoor visual inspection, *etc*. [5]. Researchers and manufacturers have recently developed a new generation of image sensors and new techniques that make it possible to increase the typical 60 dB range for a CCD sensor to 120 dB. Reference [6] reports several techniques to expand the dynamic range by pre-estimation of the sensor response curve. Reference [7] proposes to combine several RGB images of different exposures into one image with greater dynamic range. A US patent [8] claimed a CCD which reduces smear, thus providing greater dynamic range. Reference [9] reported a technique to convert a linear response of a CMOS sensor to a logarithmic response, and [10] proposed attaching a filter to the sensor to attenuate the light received by the pixels following a fixed pattern; after that, the image is processed to produce a new image with a greater dynamic range.

Although great progress has been made in the last decade concerning CMOS imaging, logarithmic response shows a large fixed pattern noise (FPN) and slower response time for low light levels, yielding limited sensitivity [11]. The main disadvantage of using a fixed logarithmic curve is that it reduces the contrast of the image as compared to a linear response, so that a part of the scene information is lost, as seen in Figure 2(b).

## 2. The Lin-Log CMOS Sensor

The work described in this paper concerns the development of a novel method which combines different algorithms to adjust the parameters which control the response curve of a Lin-Log CMOS sensor in order to increase its yield in HDR scenes.

LinLog™ CMOS image technology was developed at the Swiss Federal Institute of Electronics and Microtechnology (Zurich, Switerland). A LinLog CMOS sensor presents a linear response for low light levels and a logarithmic compression as light intensity increases. Linear response for low light levels assures high sensitivity, while compression for high light levels avoids saturation.

**Figure 3.**
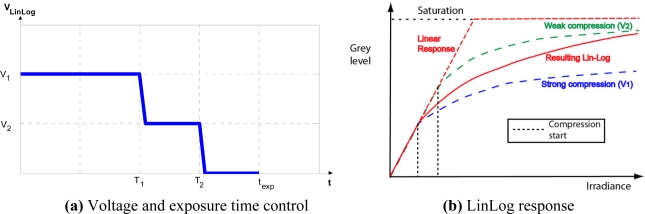
Response control for a LinLog CMOS sensor. Source: Photonfocus AG.

The transition between the two responses can be adjusted. Special attention is required to guarantee a smooth transition between them. There are various cameras (e.g., like the MV1-D1312-40-GB-12 from Photonfocus AG equipped with the Photonfocus A1312-40 active pixel LinLog CMOS image sensor which we have used for test purposes) that use LinLog technology, and sensor response can be controlled by adjusting four parameters, hereafter designated T_1_, T_2_, V_1_ and V_2_.V_1_ and V_2_ represent the compression voltage applied to the sensor. T_1_ and T_2_ are normalized parameters, expressed as a fraction of the exposure time, and can be adjusted from 0 to a maximum value of 1; their values determine the percentage of exposure time during which V_1_ and V_2_ are applied [Figure 3(a)]. The values of these four parameters determine the LinLog response of the sensor [Figure 3(b)]. Note that the final LinLog response is a combination of: (1) the linear response, (2) the logarithmic response with strong compression (V_1_) and (3) the logarithmic response with weak compression (V_2_) [Figure 3(b)]. These responses are combined by adjusting T_1_ and T_2_ values.

We have taken control characteristics of the LinLog CMOS sensor to develop a real-time image improvement method for high dynamic range scenes. This is made up of three different algorithms: (1) an algorithm to control the exposure time, (2) an algorithm to avoid image saturation, and (3) an algorithm to maximize the image entropy.

## 3. Exposure Time Control Module

**Figure 4.**
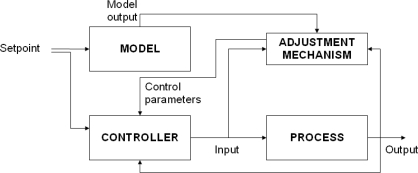
Model for an adaptive controller.

We have developed a module that controls the exposure time in order to assure that the average intensity level of the image tends to a set value (usually near the mean of available intensity levels), thus offering automatic correction of the deviations caused by variable lighting conditions in the scene. For this purpose we have implemented an Adaptive Proportional-Integral-Derivative controller (APID) which compensates non-linear effects at the time of image acquisition, by adjusting the exposure time as scene lighting conditions vary. An adaptive control system [12] measures the process response, compares it with the response given by a reference process and is capable of adjusting process parameters to assure the desired response as shown in Figure 4.

**Figure 5.**
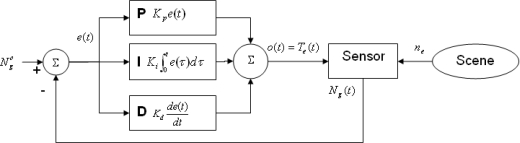
PID **controller** scheme.

In our case, the process that is controlled is acquisition of an image by a LinLog CMOS sensor. The output is the intensity level of that image. To quantify this level we use Equations (1) and (2):

(1) $$N_{g}=\frac{\left(\sum_{i=0}^{D-1}\left(i+1\right)H\left(i\right)\right)-N}{N\left(D-1\right)}$$

(2) $$D=2^{d}$$

where *N_g_* [0,1] represents the intensity level, *d* is the number of bits per pixel and *D* is the number of gray levels. *H*(*i*) represents the i-th histogram entry and *N* the number of pixels in the image. *N_g_* is used as an input parameter for a Proportional-Integral-Derivative (PID) controller [13–15] which controls some camera parameters (see Figure 5), as shown in Equations (3) and (4).

(3) $$o\left(t\right)=K_{p}e\left(t\right)+K_{i}\int_{0}^{t}e\left(t^{\prime }\right){\mathrm{dt}}^{\prime }+K_{d}\frac{de\left(t\right)}{dt}$$

(4) $$e\left(t\right)=N_{g}^{o}-N_{g}\left(t\right)$$

where *o*(*t*) is controller output (exposure time) and *e*(*t*) the error value (difference between real -*N_g_*(*t*)- and set - $N_{g}^{o}$- intensity levels). *K_p_*, *K_i_* and *K_d_*, are gain values for the PID action. These gain values can be adjusted by means of either empirical or specific methods [16]. For implementation of the controller, there are a number of requirements to be considered:

- The integral action must be set to a reference value.
- The time taken to calculate integration error must be limited.
- The integral term must not continue to increase once the maximum or the minimum output values have been reached.

The next step is to model the process. Usually, if we obviate the LinLog effect, the total number of electrons for every pixel in the image can be defined as Equation (5):

(5) $$n_{e}=\frac{P_{s}A_{p}T_{e}}{hc}\lambda \eta \left(\lambda \right)$$

where *n_e_* is the number of electrons per pixel, *A_p_* is the pixel area, *T_e_* is the exposure time, *P_s_* is the power radiated onto the pixel area, *h* is the Plank constant, *c* is the light speed and *η*(*λ*) is the quantum efficiency. The conversion of electrons to an output voltage and then to a quantification level in the A/D converter depends on sensor amplification, but it can be modelled by a constant, *k*, resulting in *N_c_* *= n_e_/k*. As we can see, then, the only time-dependant variables are *P_s_* and *T_e_*, *T_e_* being the output to be controlled Equation (6):

(6) $$N_{c}\left(t\right)=CP_{s}\left(t\right)T_{e}\left(t\right)$$

The gain of the process can thus be defined as *CP_s_*(*t*) (where *C* represents the constants of Equation (5). PID parameters *K_p_*, *K_i_*, and *K_d_* are functions of this gain [10], so temporal variation of them is related to gain variation. We can use Equation (7) to estimate the gain variation every time the system feeds back, *τ* being the feedback period. From now on, to simplify following Equations (7), (8) and (9) we will use *G*(*t*) for *CPs*(*t*):

(7) $$\Delta_{t}G\left(t\right)=G\left(t\right)-G\left(t-\tau \right)=\frac{N_{c}\left(t\right)}{T_{e}\left(t\right)}-\frac{N_{c}\left(t-\tau \right)}{T_{e}\left(t-\tau \right)}$$

In time *t* we use *G*(*t-τ*) to calculate PID parameters, since *G*(*t*) cannot be calculated until *T_e_*(*t*) is known. To calculate *G*(*t*) we use *N_c_*(*t-τ*), *N_c_*(*t-2τ*), *T_e_*(*t-τ*) and *T_e_*(*t-2τ*), justified by Equation (8):

(8) $$\tau \to 0\Rightarrow \Delta_{t}G\left(t-\tau \right)\approx \Delta_{t}G\left(t\right)$$

Only parameters *K_p_* and *K_i_* are updated, as shown in Equation (9), where *α*, *β*, *M* and *l* are parameters to be fixed by the designer:

$$\Delta_{t}K_{p}=\{\begin{array}{cc} \alpha \Delta_{t}G & \text{if}\Delta_{t}G\in \left[-l,l\right]\\ 0 & \text{otherwise} \end{array}$$

(9) $$\Delta_{t}K_{i}=\{\begin{array}{cc} \beta \Delta_{t}G & \text{if}\Delta_{t}G\in ([-M,-l)\cup (l,M])\\ \text{sgn}(\Delta_{t}G)\cdot \beta M & \text{if}\Delta_{t}G\in ((-\infty ,-M)\cup (M,\infty ))\\ 0 & \text{otherwise} \end{array}$$

**Figure 6.**
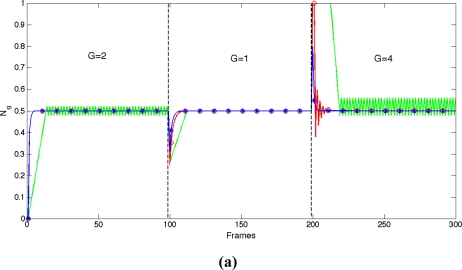

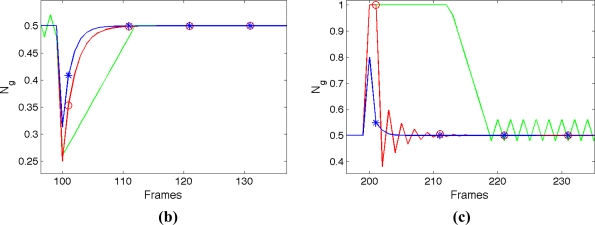
Performance of **PI**, APID and *Incremental* controllers.

Figure 6(a) shows the response of the proposed APID controller—“*”, blue- *versus* other controllers mentioned in the references: Proportional-Integral (PI) [17,18]—“o” red- and a controller designated *Incremental*, based on increments that are proportional to the error (“-” green) [19]. In order to compare the responses, the PI and APID controllers were configured with the same constants (*K_p_* *= 0.01*, *k_i_* *= 0.4* and *K_d_* *= 0*) and the parameters of the *Incremental* controller were adjusted to achieve the best combination of speed and stability. Even so, unwanted oscillations may appear and this has proven to be the slowest of the three controllers. During the first frames the PI and APID controllers showed the same response because they had the same initial configuration.

Figure 6(b) shows the performance details of the controllers *versus* a decrease of the process gain *ΔG =* −*1*. The APID controller increases its internal gain, producing faster performance (*l = 0.1*, *M = 0.6*, *α = 0.01*, *β = 0.1*).

Figure 6(c) shows the performance details of the controllers *versus* an increase of the process gain *ΔG = 3*. The APID controller reduces its internal gain to prevent overshoot and oscillations and keeps speed. To the contrary, the PI controller is unable to prevent overshoot. Table 1 shows measurements for time response, overshoot and oscillation of controller responses shown in Figure 6.

## 4. Saturation Control

We can detect image saturation when saturation $N_{S}^{B}$ width (see Equation (10)) reaches a given value. To reduce saturation we increase the voltage values that control the LinLog compression effect:

(10) $$N_{S}^{B}=\frac{H\left(D-1\right)}{N}$$

where *H*(*D*−1) is the (*D*−1)-th entry for the image histogram, *H*.

**Figure 7.**
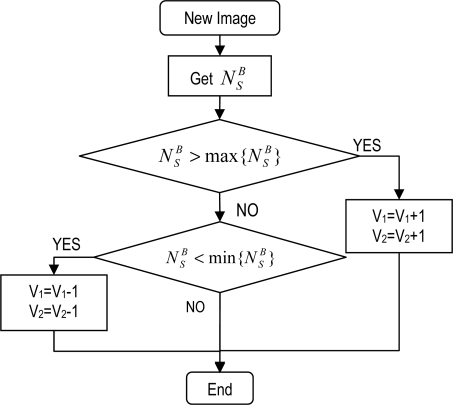
Algorithm for saturation control in a LinLog CMOS sensor.

Figure 7 shows the algorithm for saturation control. This measures the saturation width given by Equation (10). V_1_ and V_2_ are increased or reduced depending on whether the measured value is greater or smaller than the set value.

**Figure 8.**
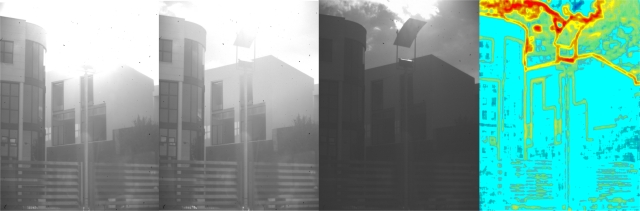
Different images taken from a scene with increasing values of V_1_ and V_2_ from left to right. The last image on the right shows the local entropy map of the image on its left (maximum values in red, minimum values in blue).

Figure 8 shows the effect of varying V_1_ and V_2_ values.

## 5. Entropy Maximization

The concept of information entropy describes how much randomness (or uncertainty) there is in a signal or an image; in other words, how much information is provided by the signal or image. In terms of physics, the greater the information entropy of the image, the higher its quality will be [20].

Shannon’s entropy (information entropy) [21] is used to evaluate the quality of acquired images. Assuming that images have a negligible noise content, the more detail (or uncertainty) there is, the better the image will be (the entropy value for a completely homogenous image is 0). That is, without analyzing the image content, we assume (for two images obtained from an invariant scene) that the richer the information, the greater will be the entropy of the image.

**Figure 9.**
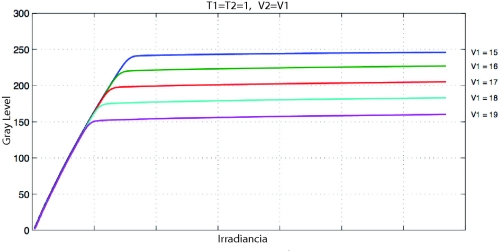
By increasing V_1_ and V_2_ values we can increase the compression for high intensity levels (values for MV1-D1312 camera). Source: Photonfocus AG.

**Figure 10.**
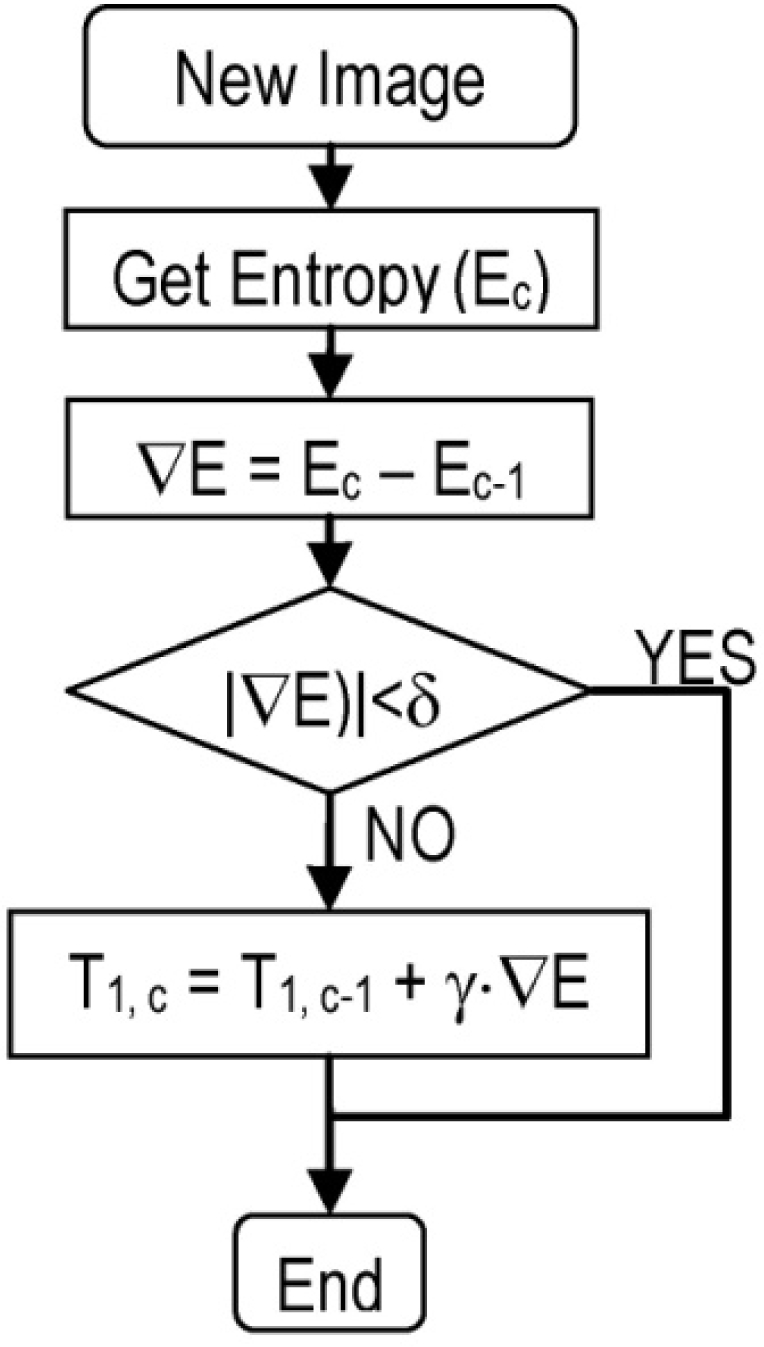
Algorithm to adjust T_1_ value for a LinLog sensor (where “c” is current iteration, “c − 1” is the result of previous iteration (E_c − 1_ = 0 when algorithm starts), “δ” is the condition for entropy to stop the algorithm) and “γ” is the step size.

The response curves, as shown in Figure 9, cause a loss of resolution in the bright areas of the image. Moreover, although the algorithm presented in Section 4 prevents saturation it can reduce the contrast in dark areas of the image. To deal with this problem, we have developed an algorithm (see Figure 10) that maximizes the entropy (Equation (11)) of the image.

**Figure 11.**
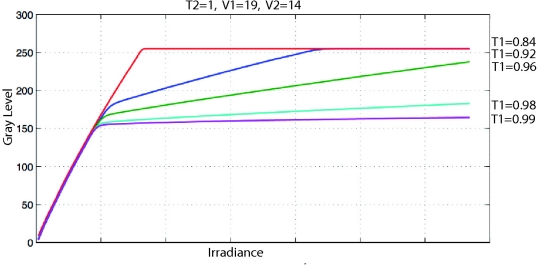
Reduction of T_1_ value permits a more linear response for high level illumination, although the slope of the response is smaller (MV1-D1312 camera). Source: Photonfocus AG.

For this purpose we adjust T_1_ (Figure 11) to produce light linearization for the high irradiance response curve. To reduce the complexity of the algorithm, T_2_ is set to a maximum and remains constant:

(11) $$E\left(X\right)=\{\begin{array}{cc} -\sum_{i=1}^{D}p\left(x_{i}\right){\text{log}}_{2}\;p\left(x_{i}\right) & p\left(x\right)\neq 0\\ 0 & p\left(x\right)=0 \end{array}$$

where *E*(*X*) is the entropy of the image *X* and *p*(*x_i_*) is the probability mass function of the grey level *x_i_*. The entropy is a measure of the information contained in the image. In this paper, we assume that an image of a scene has been taken with optimum sensor configuration when its maximum entropy has been reached.

The main difficulty in developing an algorithm for entropy maximization [22] lies in the fact that it is not possible to fix a target entropy *a priori*, since this value depends on the scene. As shown in Figure 10, the algorithm is local maximizer-like [23] and has desirable properties for our purpose. The most desirable property in this case is robustness; the control method based on the conjugated gradient ensures an asymptotic tendency toward the nearest local maximum with δ accuracy, and furthermore is an easy method to implement. For this reason it has already been used to control parameters of a camera sensor [24]. In other cases, non-adaptive PI controllers have been used [17,18], but they are not robust in non-linear systems. The second-order Taylor polynomial expansions of the gradient method (Newton, Levenberg-Marquard, *etc.*) [25] present a higher convergence speed but are more prone to instabilities [26]. When the scene changes, the gradient direction may also change and, in a first step, the algorithm will get the maximization direction wrong, but this will be corrected in the next step. Therefore, the algorithm’s performance is robust if we assume that scene variation is slower than the period between algorithm steps.

**Figure 12.**
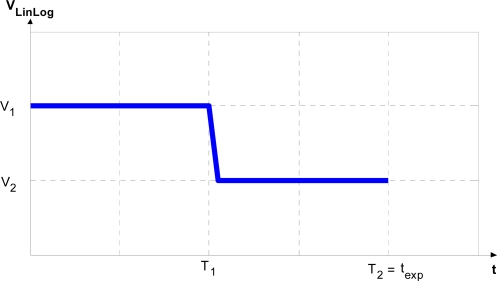
Algorithm adjusts V_1_, V_2_ and T_1_.

**Figure 13.**
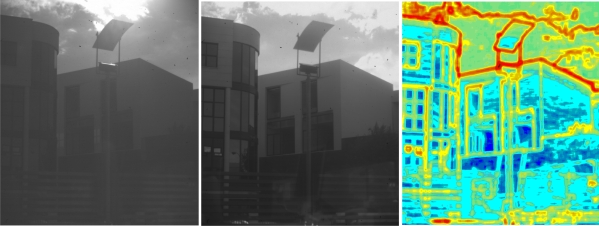
Image captured with the same values for V_1_ and V_2_, but with APID adjustment of T_1_ (centre). The local entropy map of the central image is shown on the right. Note that in the middle image the details in the scene are well defined with no loss of contrast, as compared with the image on the left.

The execution of the algorithm will be stopped when a minimum variation in entropy, δ, is reached. To avoid undesired oscillations of image contrast γ needs to be small. Even so, the algorithm developed here shows a quick response when working in continuous grabbing mode. We can see how V_1_, V_2_ and T_1_ are adjusted (Figure 12) and the improvement provided by the algorithm developed (Figure 13).

## 6. Results and Discussion

The proposed method comprises three algorithms to control the sensor response: the algorithm that controls exposure time is executed simultaneously with the other two—the algorithm that controls image saturation (adjusts V_1_ and V_2_) and the algorithm that maximizes the image entropy (adjusts T_1_); these last two algorithms are executed consecutively. Hence, the total time for the adjustment process will be the maximum of: (1) exposure adjustment time and (2) the sum of the times of the two algorithms for controlling the LinLog parameters. The time exposure controller takes less than 10 frames to respond to the step inputs (Figure 6) with the sensor running at 27 fps, which makes it suitable for use in real-time outdoor vision (Figure 15).

To gauge the performance of the image saturation control and the entropy maximization algorithms, an experiment has been designed to determine both the response speed and the resulting image quality. For this purpose:

(a) The sensor response has been modelled *versus* the irradiance, by approximating it to the curves provided by the manufacturer (Figures 9 and 11), as seen in Equations (12) and (13):

(12) $$Nc=\{\begin{array}{cc} C^{l}I & I<I_{V}\\ \text{min}\left\{C^{l}I,C_{1}^{g}+C_{2}^{g}\;{\text{log}}_{10}\left(I\right),D\right\} & \text{otherwise} \end{array}$$

where *N_c_* is the grey (unitless) output level for each pixel and *I* is the effective irradiance on the pixel (we assume *I = I_0_* *− I_r_*; *I_r_* is the real irradiance and *I_0_* the minimum irradiance detectable by the sensor. *I_0_* will depend on the configuration of the sensor exposure time parameter; in our experiment we assume a fixed exposure time and *I_0_* *= 0*). The other values of the above mentioned expression depend on the configuration of the Lin-Log parameters of the sensor and were obtained by approximating the curves and data provided by Photonfocus in the *User Manual* of the camera used in the tests (MV1-D1312):

(13) $$\begin{array}{l} C_{2}^{g}=\frac{D-C^{l}I_{v}}{{\text{log}}_{10}\left(I_{T}\right)-{\text{log}}_{10}\left(I_{v}\right)},\;\;C_{1}^{g}=D-C_{2}^{g}\;{\text{log}}_{10}{\left(I_{T}\right)}_{,\;\;}I_{T}=\left(I_{h}^{1}-I_{h}^{2}\right)T+I_{h}^{2}\\ \;\;\;\;\;\;\;\;\;\;\;\;\;\;\;\;\;\;\;\;\;\;\;\;\;\;\;\;\;\;\;\;\;I_{v}=\frac{D-C_{1}I_{h}^{1}}{C^{l}-C_{1}},\;\;I_{h}^{1}=10^{\frac{1}{2}\left(V-7\right)},I_{h}^{2}=10^{\frac{1}{2}\left(V-8\right)} \end{array}$$

where *C^l^* = 2.55 m^2^/*W* and *C*_l_ = 10,911 m^2^/*W*. V is the parameter V_1_; it is assumed that V_2_ = V_1_ − 5, and T corresponds to the parameter T_1_ (it is assumed that T_2_ = 1). According to this model, the sensor has a dynamic range of 120 dB when configured in maximum compression mode (V = 19 and T = 1) and its response is linear when there is no compression (V = 14 and T = 0).

**Figure 14.**
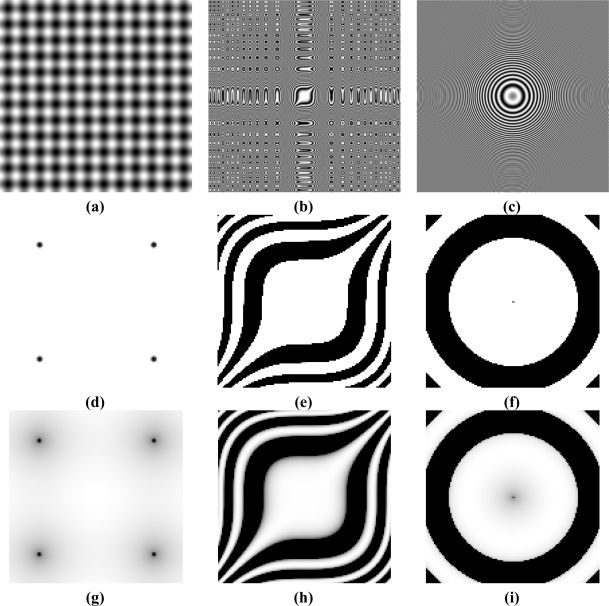
Synthetic scenes **(a, b, c)**—corresponding to patterns 1, 2, and 3 of Table 2 and details of images obtained by the CMOS sensor working in linear mode **(d, e, f)** and in LinLog mode **(g, h, i)**, with LinLog parameters adjusted by the proposed method.

(b) Three synthetic scenes have been generated with patterns $I_{p}^{i}\left(x,y\right)$ with *i* ∈ [1,2,3] as illustrated in Figure 14. Each value of the pattern $I_{p}^{i}\left(x,y\right)$ represents the irradiance at the point (x, y) (Table 2). The dynamic range of each scene is shown in Table 2.

The characteristic entropy value has been calculated for each one of the scenes (Table 3). The entropy value (defined as pattern entropy) has been calculated using Equation (11). Pattern data are expressed in double precision floating point format.

**Figure 15.**
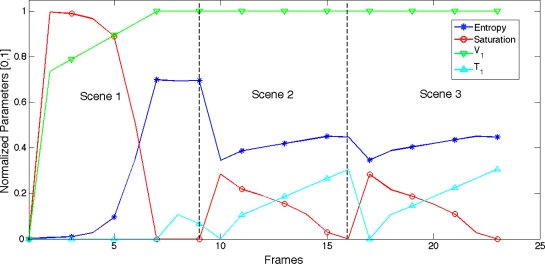
Adjustment of LinLog parameters as scene changes. For scene 1 the saturation control algorithm increases V_1_—from start-up settings—until saturation disappears; for scenes 2 and 3 saturation is kept under control, while T_1_ is adjusted to maximize the entropy.

(c) The synthetic scenes have been consecutively processed with the camera model to evaluate the temporal performance of the system both in the start-up and when responding to the scene changes. Figure 15 shows the time evolution of the LinLog parameters produced by the saturation control and entropy maximization algorithms.

To use a sensor’s model together with synthetic scenes allowed defining a metric to quantify the retained entropy, which resulted on reliable yield evaluation, useful for further comparisons.

Figure 14 shows the synthetic scenes used to test the proposed method, together with images obtained when the scenes have been processed by the LinLog CMOS sensor’s model [Equations (12,13)]. Figure 15 shows how the control method adjusts LinLog parameters as different scenes are presented to the sensor.

Table 3 shows the numeric results of the experiment. Besides the response time, it shows the recovered entropy once the synthetic scenes are processed by: (1) a model of a typical linear CMOS sensor (DR of 60 dB)—adjusted so that *I_0_* corresponds to grey level 0—and (b) the proposed LinLog model with its parameters adjusted using the proposed control method.

The pattern entropy values will be reduced during the digital image generation process. Hence, the pattern entropy percentage retained in the acquired image of the scene provides an objective measurement of the goodness of the sensor’s parameters control process. The higher the entropy of the acquired image, the better the control process is, as there is more scene information. As we can see in Table 3, with the proposed method at least 67% of pattern entropy can be retained in the images.

**Figure 16.**
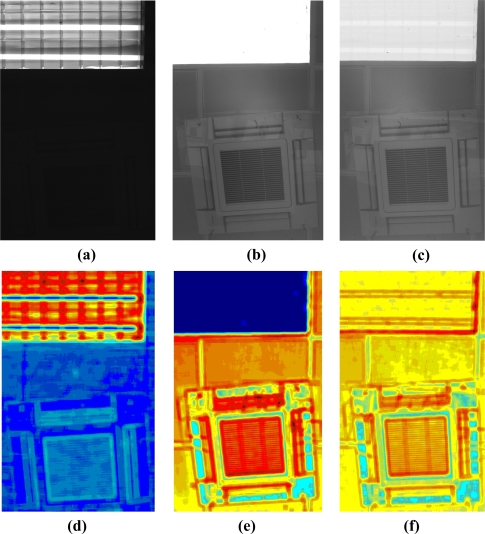
Image **(a)** was acquired with the image sensor working in linear mode; exposure time was adjusted to capture details of the lighting source; Image **(b)** was also acquired with the image sensor working in linear mode, but here the exposure time was adjusted to capture details out of the lighting source; in this case, the details from the lighting source disappear; Image **(c)** was acquired in LinLog mode automatically adjusted using the proposed method; as we can see, both details of the source and of the scene are retained in the image; Local entropy maps **(d)**, **(e)** and **(f)**, which correspond to images (a), (b) and (c) respectively, help give an idea of the extent of the improvement in image (c).

Images (a) to (f) in Figure 16 show how the proposed method performs over a very high dynamic range scene (the ceiling of our lab, with a powerful lighting source).

**Figure 17.**
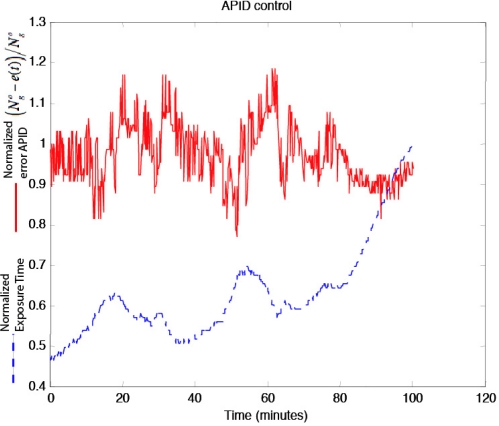
Variations of the APID controller output error (red) and the exposure time (blue)—normalized—for the system in outdoor use. There are various causes of variations in short time periods (scene changes, clouds, *etc.*). The reason why the exposure time shows a rising trend is that the control is displayed in a run-up to sundown.

Figure 17 shows how exposure time was controlled by the APID controller for a period of almost two hours between 4:30 and 6:10 pm on a windy day with clouds crossing the camera field of view (producing illumination changes), to acquire images from the scene shown in Figure 13. Sunset lasts from 5:40 until 6:10 pm.

## 7. Conclusions

This paper presents a reliable method for optimizing LinLog CMOS sensor response and hence improving images acquired from high dynamic range scenes. Adaptation to environment conditions is automatic and very fast.

The implementation has been divided into three algorithms. The first makes it possible to control the exposure time by using an Adaptive PID (APID) controller; the second controls image saturation through appropriate compression of the response curve for brilliant scenes and the third provides entropy maximization by slightly linearizing the response curve for high scene irradiance.

The simplicity of the control algorithms used in this method makes the computational cost of the processing needed to calculate the image parameters (histogram-based descriptors) negligible; therefore the computational cost of implementing the presented method practically coincides with the cost of calculating the histogram. As Table 3 shows, the control takes up less than eight frames with high quality images.

The method proposed in this paper has been implemented using NI LabVIEW [27], resulting in: (1) high-level hardware-independent development; (2) rapid prototyping due to the use of libraries (Real-Time, PID and FPGA libraries); and (3) rapid testing of the control application.

The hardware used to implement the system consisted of a Real-Time PowerPC Embedded Controller (cRIO-9022) and a reconfigurable chassis based on a Virtex-5 FPGA (cRIO-9114) from National Instruments. The chosen system permits deterministic control and real time execution of applications. The control system and the camera to be easily connected thanks to an Embedded PowerPC with GBit Ethernet and RS232 ports.

## Figures and Tables

**Table 1.** Metrics for quantifying the controllers response shown in Figure 6. Settling time is a metric, expressed in frames, which measures the run-time until the error is lower than 2%. Overshoot measures the difference between the maximum response value and the reference level. Stationary oscillation measures the amplitude of non-attenuated oscillation. The last two metrics evaluate the robustness of the controllers and are expressed as a percentage of the reference level $\left(N_{g}^{o}\right)$.

	**Incremental**			**PI**			**APID**		
Δ ***G* (t)**	0⇨2	2⇨1	1⇨4	0⇨2	2⇨1	1⇨4	0⇨2	2⇨1	1⇨4
**Settling time**	--	13	--	5	8	12	5	6	4
**Overshoot**	4	0	4	0	0	24	0	0	0
**Stationary oscillation**	4	0	16	0	0	0	0	0	0

**Table 2.** Patterns for synthetic scenes generation.

**Scene**	**Pattern, *I_p_(x,y)***	**DR(dB)**
**1**	10^5^*POS*(cos(x^2^)+cos(y^2^))	104
**2**	5·10^5^*POS*(cos(x^3^+y^3^))	116
**3**	5·10^5^*POS*(sin(x^2^+y^2^))	116

	$\mathrm{POS}\left(f\right)\{\begin{array}{cc} f & f>0\\ 0 & \text{otherwise} \end{array}$	

**Table 3.** Performance measurement illustrated in the graphs of Figure 15. F (fast) corresponds to the configuration (γ = 0.04 and δ = 0.04) and P (precise) to the configuration (γ = 0.01 and δ = 0.01).

**Scene**	**Pattern Entropy**	**Lin-Log**						**Lineal**	
		**Time (frames)**		**Retained Entropy**		**Retained Entropy(%)**		**Retained Entropy**	**Retained Entropy(%)**
*Control Mode (Fast/Precise)*		*F*	*P*	*F*	*P*	*F*	*P*		
1	7.90	8	8	5.32	5.33	67.35	67.46	0.08	1.00
2	4.86	6	27	3.42	4.38	70.42	71.57	1.01	20.79
3	4.58	6	27	3.42	3.48	70.50	71.62	1.01	20.77
